# COVID-19: the androgen hypothesis

**DOI:** 10.1186/s12301-020-00075-0

**Published:** 2020-09-22

**Authors:** Jeff John, Ken Kesner

**Affiliations:** Division of Urology, Department of Surgery, Frere Hospital and Walter Sisulu University, East London, 5200 South Africa

On December 31, 2019, the World Health Organisation (WHO) China Office was informed of patients presenting with pneumonia of unknown aetiology in Wuhan City in the Hubei Province of China. Twelve days later, China revealed the genetic sequence of the causative agent, a novel virus which the WHO named SARS Coronavirus 2 (SARS-CoV-2) and the resulting pneumonia Coronavirus Disease 2019 (COVID-19) [1]. At the time of writing, there have been 14,530,395 reported cases and 606,760 deaths (6.26%) worldwide. The African continent, home to 17% of the world’s population, currently accounts for just 4.9% of cases and 2.5% of deaths [2].

While our understanding of the nature and pathogenesis of the novel SARS-CoV-2 is rapidly evolving, there is as yet no known cure and a possible vaccine is still a work in progress. In the meantime, many hypotheses have been proposed in the hope of alleviating the severity of the disease or better still, neutering it.

One such hypothesis by Montopoli and colleagues looks into the relationship between androgens and SARS-CoV-2. They propose that androgen deprivation therapy (ADT) may protect patients with prostate cancer from SARS-CoV-2 infections. The researchers extracted data from all patients with laboratory-confirmed SARS-CoV-2 infection presenting at the numerous hospitals in Veneto, one of the Italian regions most affected by the current pandemic and matched this data with a regional database of men with prostate cancer. They concluded that men with prostate carcinoma on treatment with ADT had a significant fourfold reduced risk of COVID-19 infections as compared to men who were not treated with ADT (OR 4.05; 95% CI 1.55–10.59). Of the 5273 men on ADT, only four developed COVID-19, all of whom recovered. In comparison, the database identified 37,161 men with prostate cancer not on ADT, of whom 114 men developed COVID-19 and 18 of these patients succumbed to the illness. They went on to conclude that even if men without prostate cancer, patients at increased high risk of developing COVID-19 could take ADT for a limited period of time to prevent infection, while those who become infected could take ADT to reduce the severity of the symptoms [3]. The dataset is limited, but the hypothesis is definitely intriguing.

A scientific basis for the hypothesis that ADT may protect against COVID-19 looks at the biology of how SARS-CoV-2 enters a human cell. There are three or four viral proteins on the SARS-CoV-2 membrane, i.e., spike (S), membrane (M), envelope (E) and nucleocapsid (N) proteins. The spike (S) protein of the virus binds to angiotensin-converting-enzyme 2 (ACE-2), and this will allow the virus to enter and infect the host cell provided the viral spike protein is first primed by a cellular protease called transmembrane protease serine type II (TMPRSS2) [4, 5]. TMPRSS2 cleaves the spike protein to S1 and S2. S1 initiates binding to the host cell receptor, while S2 initiates viral and cellular membrane fusion [6]. TMPRSS2 is also believed to cleave ACE-2 for augmented viral entry [7]. Once primed, a large load of virus enters the host cell. TMPRSS2 is expressed in prostate epithelial and cancer cells and also shows low-level expression in the lungs, colon, liver, kidney and pancreas [8]. In the prostate, TMPRSS2 is expressed in an androgen dependent manner but whether TMPRSS2 expression in the normal human lung is also regulated by androgens remains unclear. [9] If it is found that TMPRSS2 in lung epithelium is indeed under androgen regulation, then lowering testosterone with ADT may be an option to lower the expression of TMPRSS2, impede viral entry, and reduce the severity or duration of COVID-19. A clinical trial to test the effect of reducing testosterone in COVID-19 patients is set to begin at three Veterans Affairs hospitals in New York City, Los Angeles, and Seattle. In this proposed double-blind randomised controlled trial, researchers will recruit 200 hospitalised male patients who will be randomised to receive a placebo or one dose of degarelix, a FDA-approved drug that rapidly reduces testosterone to castrate levels [10].

The possible role of in COVID-19 poses some more intriguing questions. Epidemiological data show increased severity of COVID-19 in the male population. Despite similar infection rates in both genders, males are more likely to develop complications, be hospitalised, require ventilatory support and have a poorer clinical outcome than females [3]. In Italy, once the global epicentre of the disease, for every ten female deaths related to COVID-19, there were 24 male deaths related to the disease [11]. This gender-based disparity was also observed during the SARS outbreak and not unique to COVID-19. Is the upregulation of TMPRSS2 by androgens responsible for the increased severity of the disease in male patients? Why then is the younger male population, who are presumed to have increased testosterone levels, relatively spared from severe disease? As men age, plasma testosterone levels fall and this decline in testosterone can cause a reduction in respiratory muscles activity, strength and exercise capacity [12]. Could this be one of the factors accounting for increased severity of disease in older men? Androgens are immunosuppressive targeting many arms of the immune system and act to dampen the immune response [13]. Could this androgen-mediated suppression of immune reactivity and inflammation contribute to heightened COVID-19 disease severity in the male? Is the male predilection more likely due to the fact men are more likely to practice unhealthy habits, than women? More than one-third (35%) of men in the world smoke, while just over 6% of women do [14]. Men also outnumber women four to one in weekly episodes of heavy drinking [15]. Furthermore, pre-existing comorbid conditions, known to be risk factors for disease severity of COVID-19, are more common among men. [11] Is the decreased disease severity in women due to the protective characteristics of oestrogen? In animal experiments, oestrogen treatment has been shown to significantly reduce the inflammatory reactions and decreases virus titers, thereby reducing mortality [16]. A phase II clinical trial is underway to establish if a 7-day course of estradiol delivered in a transdermal patch in COVID-19 or presumptive positive patients will be safe and will reduce symptom severity in adult men and older women when given prior to intubation [17].

While the association between androgens and COVID-19 is intriguing, we must also recognise that papers and studies on the SARS-CoV-2 will continue to flood in during the pandemic, both from our own continent and beyond. As clinicians, we must ensure they are carefully scrutinised and theories proven by larger clinical trials before putting any recommendation into practice. For the time being, it is worthwhile asking, should we be measuring serum testosterone levels in a COVID-19 positive patient?

## Data Availability

Not applicable.

## Abbreviations

WHO: World Health Organisation
SARS: severe acute respiratory syndrome
SARS-CoV-2: SARS Coronavirus 2
COVID-19: Coronavirus disease 2019
ADT: androgen deprivation therapy
ACE-2: angiotensin-converting-enzyme-2
TMPRSS2: transmembrane protease serine type II
OR: odds ratio
